# Predictors of Mortality in Acute Myocardial Infarction Patients With Systemic Lupus Erythematosus

**DOI:** 10.7759/cureus.79578

**Published:** 2025-02-24

**Authors:** Etuk Aniekeme, Bruno De Souza Goncalves, Komal Sodhi, Carlos Rueda Rios, Ellen Thompson

**Affiliations:** 1 Department of Cardiology, Marshall University Joan C. Edwards School of Medicine, Huntington, USA; 2 Department of Surgery, Marshall University Joan C. Edwards School of Medicine, Huntington, USA; 3 Department of Biomedical Sciences, Marshall University Joan C. Edwards School of Medicine, Huntington, USA

**Keywords:** acute myocardial infarction, healthcare, mortality, predictors, systemic lupus erythematosus

## Abstract

Introduction

Acute myocardial infarction (AMI) remains a leading cause of mortality globally, with systemic lupus erythematosus (SLE) posing additional risks for adverse outcomes.

Methods

This retrospective cohort study utilized data from the Healthcare Cost and Utilization Project National Inpatient Sample from 2016 to 2020 to investigate predictors of mortality among hospitalized AMI patients, stratified by SLE status.

Results

A total of 81,935 patients were included, with variables analyzed using multivariate logistic regression. Among SLE patients, being female, aged ≥65 years, and having a prolonged hospital stay >5 days were associated with higher mortality. Elective admissions were protective while non-Hispanic Black SLE patients showed reduced odds of mortality compared to their non-Hispanic White counterparts. Among non-SLE patients, predictors of increased mortality included being non-Hispanic Black and having prolonged hospital stays. Private insurance coverage was associated with lower mortality in this group.

Conclusion

These findings highlight critical patient- and hospital-related factors influencing mortality in AMI patients with and without SLE. Targeted strategies focusing on early recognition, effective interventions, and reducing healthcare disparities are essential to improve outcomes in this population.

## Introduction

Acute myocardial infarction (AMI) is a leading cause of mortality worldwide, responsible for 50% of sudden cardiac deaths [1,2]. It is primarily characterized by acute coronary artery occlusion, resulting in myocardial ischemia [1]. AMI predominantly occurs in the left anterior descending coronary artery or even in the left main coronary artery, leading to the development of heart failure and death if not treated [3-5]. Different risk factors are correlated with AMI, including genetics, hypertension, dyslipidemia, diabetes, smoking, and obesity. Additionally, an increase in the incidence and mortality of patients with AMI has been associated with immune system diseases such as systemic lupus erythematosus (SLE) [6]. SLE is a multisystem autoimmune disease characterized by excessive malfunctioning of immune cells that react with self-antigens and immune-complex depositions that can result in organ damage [7]. Clinical manifestations are heterogeneous and include the skin, musculoskeletal system, central nervous system, and cardiovascular system [8]. Notably, approximately 50% of patients with SLE will experience comorbid cardiovascular diseases such as endocarditis, valvular disease, pericarditis, myocarditis, and myocardial infarction [9-11].

Heart disease represents the main cause of premature mortality in SLE patients, in which dysregulation of the immune system leads to cardiac damage due to a combination of inflammation and oxidative stress [10,12]. Chronic systemic inflammation, as well as the synthesis of various autoantibodies and immune complexes coupled with profound innate and adaptative immune dysregulation, accelerates cardiovascular damage [13]. Alterations in molecular pathways that regulate nucleic acid degradation, innate immune signaling and lymphocyte activation thresholds are mechanisms that determine SLE susceptibility. Nucleic acid-containing immune complexes are modulators of the innate immune response through their capacity to access and activate receptors of immune response signaling [14]. Innate immune responses drive the secretion of chemokines and pro-inflammatory cytokines while also facilitating the recruitment and activation of neutrophils, monocytes, and macrophages [15]. These immune cells work collectively to mediate extracellular matrix degradation or remodeling, necrotic cell phagocytosis, fibroblast proliferation, and granulation [16,17]. However, excessive ventricular remodeling can lead to alterations in ventricular size and impair cardiac function, which may ultimately result in significant adverse cardiovascular events [18]. Moreover, the autoimmune-related risk factor is the change in lipid metabolism, altering the synthesis and the levels of lipid fraction, leading to dyslipidemia that may accumulate in the heart causing an atherosclerotic environment [19]. Also, endothelial dysfunction, oxidative stress, and an increased prevalence of cardiovascular disease risk factors, such as hypertension, further exacerbate the risk. Collectively, all these mechanisms create a pro-inflammatory environment, increasing morbidity and mortality among SLE patients [13,20].

Despite advances in the management of both AMI and SLE, individuals with concomitant diagnoses remain at heightened risk for adverse outcomes [11]. Due to the few studies on AMI patients with SLE, understanding, early recognition, and effective treatment are of extreme clinical importance. Identifying predictors of mortality in this high-risk population is essential to improve clinical decision-making, optimize treatment strategies, and reduce mortality rates. Therefore, this study aims to identify patient- and hospital-associated predictors of mortality among hospitalized AMI patients, stratified by SLE status. By analyzing a large national database, we seek to determine whether these predictors differ between SLE and non-SLE patients, providing insights that may inform targeted clinical interventions and healthcare policies.

## Materials and methods

Study design, patient selection, and data collection

This retrospective cohort study utilized Healthcare Cost and Utilization Project National Inpatient Sample (HCUP-NIS) data. The HCUP-NIS is the largest publicly available, all-payer inpatient healthcare database in the United States. It contains de-identified information from over 7 million hospital stays annually, representing approximately 97% of the U.S. population and allowing for national estimates of inpatient utilization, access, charges, quality, and outcomes. The database employs a stratified sampling design, including 20% of discharges from participating hospitals, which enables generalizability to the broader U.S. population. To ensure an appropriate selection of eligible patients for the study, hospital personnel trained in the use of the database examined the HCUP-NIS data using the search period from 2016 to 2020. Specifically, patients were selected using the respective codes I-21.9 for AMI and M-32.9 for SLE, and the database's extensive range of covariates allowed for examining hospital-level predictors. All hospitalizations for AMI in patients aged ≥18 years with a diagnosis of AMI, identified using the International Classification of Diseases, Tenth Revision, Clinical Modification (ICD-10-CM; https://shorturl.at/Wo0NH), were selected. For the exclusion criteria, all patients under 18 years old and patients without AMI diagnosis were excluded from this study. Patients matching the inclusion criteria had variables collected such as age, gender, race/ethnicity, median household income national quartiles, insurance type, hospital location, admission type, length of stay, and the Charlson comorbidity index.

Statistical analysis

Appropriate weights were applied to the data to account for the complex survey design and make them representative of the general population. The chi-square test was used to compare the categorical variables between AMI patients with and without SLE. A multivariate logistics model was used to examine the factors associated with mortality among hospitalized SLE patients with AMI. Stata SE 16.1 (Stata Corp, College Station, TX, USA) was used for the analyses. All data comparisons are presented as p<0.05.

## Results

A total of 920,620 patients were initially included in this study. After stratification based on the SLE diagnosis, 81,935 patients were included for analysis. These patients were selected from the HCUP-NIS database between 2016 and 2020. Table 1 summarizes the sociodemographic, hospital-level, and clinical characteristics of hospitalized patients with AMI, stratified by the presence of SLE. Patients with SLE were significantly younger, being under 65 years old as compared to the non-SLE group (p< 0.001). The SLE group had a higher proportion of female patients (p< 0.001). Racial/ethnic distribution also differed significantly, with a higher proportion of non-Hispanic White patients in the SLE group and a lower proportion of non-Hispanic Black (p= 0.002). In addition, SLE patients were significantly covered by Medicaid (p = 0.005). Mortality rates were higher in the SLE group (p< 0.001), also the patients had longer hospital stays, staying more than five days compared to the non-SLE group (p = 0.001). The Charlson comorbidity index showed that SLE patients had lower overall comorbidity scores, with 58.64% scoring between 0 and 4 compared to the non-SLE group, whereas the latter had a higher proportion of patients with scores ≥8 (p < 0.001). The median household income, hospital region, as well as hospital location, did not show differences between SLE and non-SLE patients.

**Table 1:** Sociodemographic, hospital-level, and clinical characteristics of hospitalized patients with acute myocardial infarction by systemic lupus erythematosus diagnosis

			Likelihood Ratio Tests		
	Systemic Lupus Erythematosus (Wt %)		Chi-square	df	p-value
	Yes	No			
	n = 81935	n = 838685			
Age			11.6783	1	<0.001
Less than 65	65.16	35.65			
65 years and above	34.84	64.35			
Gender			12.4865	1	<0.001
Male	28.46	63.45			
Female	71.54	36.55			
Race/Ethnicity			11.3218	3	0.002
Non-Hispanic white	55.54	36.08			
Non-Hispanic black	7.42	15.64			
Hispanic	23.13	24.54			
Non-Hispanic other	13.91	23.74			
Median household income national quartiles			2.4321	3	0.874
Quartile 1	23.24	19.8			
Quartile 2	27.49	26.05			
Quartile 3	21.45	23.61			
Quartile 4	27.82	30.54			
Insurance type			11.1243	2	0.005
Medicare	18.85	56.14			
Medicaid	45.54	19.65			
Private/Other	35.61	24.21			
Hospital region			6.5407	3	0.435
Northeast	18.32	15.65			
Midwest	26.76	26.06			
South	37.89	34.76			
West	17.03	23.53			
Hospital location			1.5075	2	0.895
Rural	4.56	10.54			
Urban nonteaching	20.43	16.45			
Urban teaching	75.01	73.01			
Mortality			12.7645	1	<0.001
No	23.54	35.98			
Yes	76.46	64.02			
Admission type			11.5432	1	<0.001
Elective	8.65	6.54			
Non-elective	91.35	93.46			
Length of stay			11.4329	1	0.001
5 days or less	43.65	58.87			
More than 5 days	56.35	41.13			
Charlson comorbidity index			12.5632	2	<0.001
0 – 4	58.64	22.45			
5 – 7	32.42	42.09			
⩾ 8	8.94	35.46			

Table 2 shows the factors associated with mortality among hospitalized AMI patients with and without SLE. Our analysis identified several factors associated with mortality among hospitalized patients with AMI with and without SLE. Among SLE patients with a diagnosis of AMI, being female (AOR: 1.53; 95% CI: 1.02-1.86) and 65 years and above (AOR: 1.65; 95% CI: 1.20-2.74) was associated with higher odds of mortality compared to being male and younger than 65 years. Elective admission (AOR: 0.59; 95% CI: 0.39-0.85) was associated with a lower risk of mortality relative to non-elective admission. In addition, non-Hispanic blacks (AOR: 0.68; 95% CI: 0.39-0.98) had lower odds of mortality than non-Hispanic whites. Patients with a length of stay greater than 5 days (AOR: 1.75; 95% CI: 1.18-2.59) were more likely to die than those with a hospital stay of 5 days or less. Higher comorbidity scores were also associated with higher odds of mortality. Among patients without SLE, non-Hispanic blacks (AOR: 1.68; 95% CI: 1.07-2.48) reported higher mortality compared to their non-Hispanic white counterparts. Additionally, being on private insurance (AOR: 0.75; 95% CI: 0.32-0.99) was associated with lower odds of mortality relative to Medicare insurance. Other factors, such as median household income, hospital region, and hospital location, did not exhibit an association with SLE.

**Table 2:** Factors associated with mortality among hospitalized patients with acute myocardial infarction with and without systemic lupus erythematosus. SLE = systemic lupus erythematosus; AOR = adjusted odds ratio; CI = confidence intervals; model adjusted for age, gender, race/ethnicity, median household income national quartiles, hospital region, hospital location, Charlson comorbidity index, insurance type, admission type, and length of stay. The boldface indicates statistical significance.

AOR (95% CI)		
	SLE	No SLE
Age		
Less than 65	Reference	Reference
65 years and above	1.65 (1.20-2.74)	1.55 (1.27–2.67)
Gender		
Male	Reference	Reference
Female	1.53 (1.02 –1.86)	1.43 (1.02–1.68)
Race/Ethnicity		
Non-Hispanic white	Reference	Reference
Non-Hispanic black	0.68 (0.39-0.98)	1.68 (1.07–2.48)
Hispanic	1.03 (0.50-2.11)	1.45 (0.78–1.98)
Non-Hispanic other	2.32 (1.75-7.21)	1.67 (0.89–2.30)
Median household income national quartiles		
Quartile 1	Reference	Reference
Quartile 2	1.06 (0.61-1.85)	1.10 (0.68–1.79)
Quartile 3	0.74 (0.39-1.43)	0.54 (0.23–1.68)
Quartile 4	0.63 (0.31-1.25)	0.75 (0.45–1.34)
Insurance Type		
Medicare	Reference	Reference
Medicaid	0.75 (0.33-1.68)	0.45 (0.12–1.43)
Private	1.04 (0.56-1.93)	0.75 (0.32–0.99)
Other	1.02 (0.45–1.67)	1.20 (0.49–2.30)
Hospital Region		
Northeast	Reference	Reference
Midwest	1.34 (0.68–2.10)	0.68 (0.32–1.43)
South	1.68 (0.88–1.99)	1.57 (0.79–1.98)
West	1.46 (0.43–1.75)	1.58 (0.87–2.34)
Hospital Location		
Rural	Reference	Reference
Urban nonteaching	1.85 (0.63–2.55)	1.45 (0.56–2.48)
Urban teaching	1.50 (0.65–2.65)	1.10 (0.74–1.86)
Admission Type		
Non-elective	Reference	Reference
Elective	0.59 (0.39-0.85)	0.23 (0.19–0.99)
Length of stay		
5 days or less	Reference	Reference
More than 5 days	1.75 (1.18-2.59)	2.67 (2.10–3.94)
Charlson comorbidity index		
0 – 4	Reference	Reference
5 – 7	2.07 (1.25-3.43)	1.45 (0.23–2.62)
⩾ 8	2.44 (1.20-4.98)	3.03 (2.12–5.45)

## Discussion

AMI remains a major global health burden, serving as the underlying cause of the majority of cardiovascular diseases [21,22]. Despite advances in medical therapy, the premature mortality of AMI patients with SLE continues to rise, encompassing direct healthcare costs [23-25]. In agreement with the literature, the results of our present study also illustrated that the interplay between AMI and SLE has a central role in cardiac deaths. Hence, it is a reasonable strategy to investigate the factors associated with mortality among hospitalized patients with AMI, with a specific focus on individuals with and without SLE. Our data emphasize critical patient- and hospital-related predictors of mortality, showing valuable insights into this high-risk population.

It has been shown that hospitalizations due to AMI are more common in SLE women aged 18-44 than in women without SLE in the same age group [26]. Among patients with SLE, being female and aged 65 years or older was significantly associated with higher odds of mortality. The elevated risk of developing cardiovascular events and worse outcomes in SLE patients may be attributed, at least in part, to hormonal changes, especially during menopause [27]. The hormonal changes lead to a decrease in estradiol, which induces immune system modifications, including increased production of pro-inflammatory cytokines, such as interleukin 6, and tumor necrosis factor-alpha, and decreased levels of anti-inflammatory cytokines. This imbalance contributes to a persistent systemic inflammatory state, prolonged disease duration, inflammation, and autoimmunity [28]. Furthermore, SLE is characterized by continuous activation of the immune response and high circulating levels of inflammatory mediators, which not only exacerbate systemic inflammation but also lead to dyslipidemia, with elevated cholesterol levels and retention of HDL in tissues [19]. These pathological mechanisms create a pro-atherogenic environment, establishing a link between age-related atherosclerosis, SLE, and AMI. In addition, elective admissions were associated with lower mortality, showing the potential protective role of planned and timely interventions in this subgroup.

It is described that SLE can be particularly severe in certain ethnic groups [29]. Interestingly, non-Hispanic black patients with SLE were associated with lower odds of mortality compared to non-Hispanic white counterparts. Moreover, in patients without SLE, the predictors of mortality differed. Non-Hispanic Black patients had higher odds of mortality compared to non-Hispanic White patients, which may reflect disparities in healthcare access, treatment quality, or socioeconomic factors. It has been shown that there is a significant disparity in cardiovascular disease risk factors across various racial and ethnic groups [30]. Specifically, non-Hispanic Black individuals exhibited the highest rates of heart issues. This may be attributed to structural and vascular differences between black and white people, such as worse arterial stiffness and microcirculatory function as well as differences in the left ventricular structural and functional changes in response to arterial afterload [31,32]. Also, different factors related to social disparities among non-Hispanic black patients have an impact on cardiovascular outcomes such as lack of education, lower income, higher rates of unemployment, and poor living conditions. Additionally, these populations suffer from higher levels of food and housing insecurity and lack of adequate insurance coverage, all of which contribute to poorer health [32]. In addition, it is well established that health insurance is associated with increased access to medical care and helps protect against the high costs of illness [4].

Length of hospital stay (LOS) following AMI has steadily decreased due both to improved treatments and cost considerations [33]. A longer hospital stay was associated with increased mortality among SLE patients, with those hospitalized for more than five days exhibiting higher odds of mortality. This association could reflect the severity of illness or complications that necessitate prolonged hospitalization. Similarly, a higher comorbidity score was correlated with increased mortality, underscoring the compounded risk posed by multiple coexisting conditions in this vulnerable population. Interestingly, private insurance coverage was associated with lower odds of mortality, suggesting that insurance type may influence access to timely and comprehensive care, once those individuals with insurance have more physician visits and a higher prevalence of recommended preventive services. Also, the insurance type is related to assessing cardiac rehabilitation programs as well as an indicator of better quality of life.

However, the study has limitations. Cardiovascular disease-specific mortality was not assessed using the dataset; hence, all-cause inpatient mortality was explored. There is also a possibility for coding errors used to identify patient diagnoses. Moreover, establishing temporality proved challenging, as the initial diagnosis of SLE, AMI, and severity could not be determined. Also, the dataset does not include detailed clinical variables, such as inflammatory markers, plasma levels of lipid fractions in the patients with SLE in particular, the rate of prescription of lipid-lowering therapy (statins) to patients with SLE, medication adherence, and echocardiographic parameters, which could offer valuable insights. Despite these limitations, the study's primary strength lies in the use of HCUP-NIS, a robust and nationally representative dataset, which ensures the generalizability of findings to the U.S. population. Additionally, the stratified analysis based on SLE status provides valuable insight into the different predictors of mortality in this vulnerable subgroup, optimizing resource allocation, and timely interventions for AMI patients with SLE.

## Conclusions

This study comprehensively analyzes mortality predictors among hospitalized AMI patients stratified by SLE status. Our findings emphasize the importance of a multidisciplinary approach in managing AMI patients, particularly those with SLE, to improve care delivery and reduce mortality. The identification of specific mortality predictors can inform risk stratification models and improve clinical decision-making. Moreover, incorporating these insights into clinical practice has the potential to improve early recognition, optimize resource allocation, and promote timely interventions for AMI patients with SLE. Further studies are needed to explore these factors, as this will help physicians identify patients that need closer monitoring, elucidate the underlying mechanisms driving these disparities, and optimize therapeutic strategies aimed at reducing mortality in this high-risk population.
